# *Haemophilus parasuis* Encodes Two Functional Cytolethal Distending Toxins: CdtC Contains an Atypical Cholesterol Recognition/Interaction Region

**DOI:** 10.1371/journal.pone.0032580

**Published:** 2012-03-07

**Authors:** Mingguang Zhou, Qiang Zhang, Jianping Zhao, Meilin Jin

**Affiliations:** National Key Laboratory of Agricultural Microbiology, College of Veterinary Medicine, Huazhong Agricultural University, Hubei, People's Republic of China; Russian Academy of Sciences, Institute for Biological Instrumentation, Russian Federation

## Abstract

*Haemophilus parasuis* is the causative agent of Glässer's disease of pigs, a disease associated with fibrinous polyserositis, polyarthritis and meningitis. We report here *H. parasuis* encodes two copies of cytolethal distending toxins (Cdts), which these two Cdts showed the uniform toxin activity in vitro. We demonstrate that three Cdt peptides can form an active tripartite holotoxin that exhibits maximum cellular toxicity, and CdtA and CdtB form a more active toxin than CdtB and CdtC. Moreover, the cellular toxicity is associated with the binding of Cdt subunits to cells. Further analysis indicates that CdtC subunit contains an atypical cholesterol recognition/interaction amino acid consensus (CRAC) region. The mutation of CRAC site resulted in decreased cell toxicity. Finally, western blot analysis show all the 15 *H. parasuis* reference strains and 109 clinical isolates expressed CdtB subunit, indicating that Cdt is a conservative putative virulence factor for *H. parasuis*. This is the first report of the molecular and cellular basis of Cdt host interactions in *H. parasuis*.

## Introduction

*Haemophilus parasuis* (*H. parasuis*) is a small, pleomorphic, NAD-dependent member of the *Pasteurellaceae* family. This bacterium is the etiological agent of Glässer's disease in swine, which is characterized by polyserositis and arthritis [1]. In addition to Glässer's disease, *H. parasuis* causes other clinical symptoms, such as pneumonia, and colonizes the upper respiratory tract of healthy animals. This disease has increased in prevalence and severity in recent years with the adoption of new production technologies, which is one of the main causes of mortality and significant financial losses in swine industry today [2]. To date, 15 known serovars have been described, but up to 25% non-typeable are frequently recovered from clinical disease pigs according to the geographic region and the typing method [3]. However, no clear correlation between virulence and serovar has been demonstrated, for a considerable genetic heterogeneity can be still detected, not only between but also within serovars [4]–[6].

Little is known about the virulence factors specific to *H. parasuis*, and recent studies are just beginning to reveal potential components of disease-causing mechanisms. A comparison of *H. parasuis* strains suggested that certain outer membrane protein profiles may be associated with the virulence [7], although none of the proteins was further characterized. Additionally, a global search for *H. parasuis* virulence genes using differential-display RT-PCR identified relatively few promising targets [8], while other investigations assessing gene expression in vivo [9], or under in vitro growth conditions designed to mimic those encountered in vivo, identified numerous potential virulence genes, including a variety of transporters, metabolic and biosynthetic enzymes, putative cell surface proteins, and some apparent homologs of virulence genes expressed by other members of the *Pasteurellaceae* [10], [11]. Recently, *H. parasuis* was shown to induce apoptosis of epithelial cells through a lipooligosaccharide-independent mechanism, and the lipooligosaccharide of *H. parasuis* was shown to have a limited role in adhesion to newborn pig tracheal cells [12]. However, in the absence of genetic tools, the precise roles of these potential virulence factors have not yet confirmed.

The cytolethal distending toxin (Cdts) are a family of heat-labile protein cytotoxins produced by several different bacterial species including diarrheal disease-causing enteropathogens such as some *Escherichia coli* isolates, *Campylobacter jejuni*, *Shigella species*, *Haemophilus ducreyi*, and *Aggregatibacter actinomycetemcomitans* [13]–[17]. The cellular responses to DNA damage, leading to the characteristic G_2_/M cell cycle arrest, cellular distention, and nuclear enlargement were observed in intoxicated cells [14]. Indeed, CdtB exhibits limited amino acid sequence similarity with the DNase I family of proteins, and the purified CdtB exhibits very low but measurable DNase activity. When delivered into host cells by CdtA and CdtC, the active subunit CdtB is transported to the nucleus where it causes DNA damage [18]–[20]. There is also a compelling evidence that CdtB must be internalized to induce cell cycle arrest [21], [22] and function as a phosphatidylinositol 3,4,5- triphosphate phosphatase similar to that of the tumor suppressor phosphatases, PTEN and SHIP1 [23], [24]. To date, there is no report available to ascribe the specific function to Cdt in *H. parasuis*. In the present study, we firstly identified *H. parasuis* encodes two functional Cdts, and then determined what is the biological function of Cdts in *H. parasuis*.

## Materials and Methods

### Bacteria and Cell Culture

The *H. parasuis* SH0165 strain and reference strains for *H. parasuis* serovars 1–15 were maintained on Tryptic Soy agar (Difco Laboratories, Detroit) containing 10% bovine sera and 0.01% nicotinamide adenine dinucleotide (NAD) or cultured in Tryptic Soy Broth (TSB) medium (Difco Laboratories, Detroit) plus 10% bovine sera and 0.01% NAD at 37°C aerobically. A total of 109 *H. parasuis* strains were isolated between 2002 and 2010 from diseased pigs suffering polyserositis, pneumonia or septicemia in china. Identification of the isolates was carried out by biochemical tests (NAD-dependency; absence of hemolysis; urease, oxidase and catalase tests) and 16S diagnostic PCR.

The porcine alveolar macrophage cell line PAM and the human T cell leukemia cell line Jurkat E6-1 were obtained from the ATCC and cultured in RPMI 1640 supplemented with 10% FCS, 2 mM glutamine, 10 mM HEPES. The porcine kidney epithelial cell line PK-15 was obtained from the ATCC and maintained in DMEM supplemented with 10% FCS, 2 mM glutamine, 10 mM HEPES.

### Cell cycle analysis

To measure Cdt-induced cell cycle arrest, the cells (2×10^6^ cells/well) were exposed to medium or 200 µl toxin preparation (50 µg/ml) as indicated for 24 h. The cells were washed and fixed for 60 min with cold 80% ethanol. After washing, the cells were stained for 30 min with 10 µg/ml propidium iodide containing 1 mg/ml RNase (Sigma-Aldrich, St. Louis, MO). Samples were analyzed on a FACStarPlus flow cytometer. A minimum of 20,000 events was collected on each sample. For Methyl-β- cyclodextrin (mβCD) treatment, the cells were pre-incubated with mβCD (5 mM or 10 mM) for 30 min in FCS-free medium at 37°C. Cells were cooled on ice for 20 min and washed twice with cold PBS. After toxin exposure on ice for 30 min, the cells were washed five times and incubated for 24 h at 37°C in complete medium for the cell cycle analysis as described above.

### Construction of Plasmids Containing Two Cdts

The synthetic oligonucleotide primer pairs shown in Table S1 were used to amplify two *cdt* orfs. To distinguish two cdts, the pGEMCdtABC_1_ plasmid was constructed using P1/P2, which contains the front cdt, and an additional 1 kb of sequence upstream of *cdtA* and 1 kb of sequence downstream of *cdtC* gene. Similarly, the plasmid pGEMCdtABC_2_ was constructed using P3/P4, containing the post orf and an additional 1 kb of sequence upstream of *cdtC* and 1 kb of sequence downstream of *cdtA*. Individual expression plasmids were constructed for each Cdt subunit in pET28a: pET28aCdtA, pET28aCdtB and pET28aCdtC. Construction of these plasmids involved two sequential PCR. The first reaction used the primers indicated in pGEMCdtABC_1_ and pGEMCdtABC_2_, as templates. The products of the first PCR were then used as template to amplify individual *cdt* genes in each cdt orf. The resulting PCR products were then cloned in pET28a, and the plasmids were transformed into *E. coli* BL21 (DE3).

### Isolation of Recombinant CdtA, CdtB and CdtC Proteins

Cultures of transformed *E. coli* BL21 (pET28aCdtA, pET28aCdtB and pET28aCdtC) were grown in 1 L of LB broth and induced with 1 mM IPTG for 3 h. Bacteria were harvested, washed and suspended in 10 ml of 1× binding buffer (5 mM imidazole, 0.5 M NaCl, 20 mM Tris-HCl, pH 7.9). The suspension was sonicated and centrifuged at 6,000 g for 15 min. The final inclusion body pellets were suspended in 10 ml of 1× binding buffer containing 6 M urea. After incubation on ice for 1 h to dissolve the protein completely, the suspension was centrifuged at 12,000 g for 30 min. The bacterial extracts were isolated by nickel affinity chromatography, and the eluted proteins were dialysed against 10 mM Tris-HCl (pH 7.9), 100 mM NaCl, 5 mM MgCl_2_ to promote refolding.

### DNAse activity assay

DNase activity was assessed as described before [25]. Supercoiled pET28a plasmid DNA (1 µg per reaction) was incubated with various amounts of purified His-tagged CdtB protein (0–8 µg of protein per reaction).Gels were stained with ethidium bromide to view any changes in the electrophoretic mobility of the supercoiled plasmid DNA band.

### Construction of CdtB and CdtC Mutants

Mutagenesis of the *cdtB* and *cdtC* gene was performed using the Chameleon double-stranded, site-directed mutagenesis kit (Table S2) (Stratagene, Santa Clara, CA). Those amino acids of CdtB corresponding to DNase I active site residues and those amino acids of CRAC site in CdtC were chosen for mutagenesis.

The pET28aCdtB and pET28aCdtC plasmids served as the template for mutagenesis. Amplification of the mutant plasmids was conducted using PfuUltra HF DNA polymerase, construction and characterization of these plasmids was previously described. All mutants were verified by DNA sequencing. The expression of the plasmids and purification of the mutant peptides are described below.

### Cell Binding Assay

The cells were added to six well plates (1×10^6^ cells/well) and incubated for 18 h at 37°C in a moist atmosphere containing 5% CO_2_. The various concentration of purified His-tagged Cdt proteins was added to the wells. The cells were incubated on ice for 20 min, washed twice with PBS and fixed in 10% formalin for 15 min at room temperature. The cells were washed three times, and blocked with 3% BSA in PBS for 30 min, and then incubated with His-Tag monoclonal antibody diluted 1∶1000 in 3% BSA–PBS for 1 h. Unbound antibody was removed by washing three times with PBS, and incubated with a FITC-conjugated goat anti-mouse IgG for 30 min. After three washes with PBS, the cells were removed mechanically with a rubber policeman and used for flow cytometry, with data from 2×10^4^ cells being collected and analyzed in each experiment.

### Western blot analysis of CdtB

10 ml of bacterial cultures were harvested and centrifuged at 4,000 g for 15 min. The pellet was resuspended in 1 ml of cell lysis buffer (50 mM Tris-HCl (pH 8.0), 10 mM EDTA, 1%SDS, and 1×AEBSF) and whole bacterial cell lysates were obtained by sonication and subsequent centrifugation. The whole cell proteins were collected, and subjected to sterile filtration (0.22 µm). Protein concentrations were determined using the Bio-Rad protein assay.

The procedure for western blot was performed according to Zhang [26]. Gels were blotted onto PVDF membranes. Membranes were blocked in 5% skimmed milk in PBS for 1 h at room temperature and then probed with anti-CdtB mouse sera (1∶500) for 1 h at room temperature. Membranes were washed and incubated with goat anti-mouse IgG (H+L) –HRP (1∶5,000) (Southern Biotech) for 1 h at room temperature, followed by development with Supersignal west pico chemiluminescent substrate and imaged on the Image Station 2000MM (Kodak).

### Statistics

Results were assessed by Student t test for paired date. P≤0.05 was considered statistically significant (^*^, P≤0.05; ^**^, P≤0.01). In some case, the post-hoc t tests were performed after ANOVA using the Bonferroni correction to adjust for an inflated probability of a Type I error.

## Results

### *H. parasuis* SH0165 encodes two copies of Cdts

**Figure 1:**
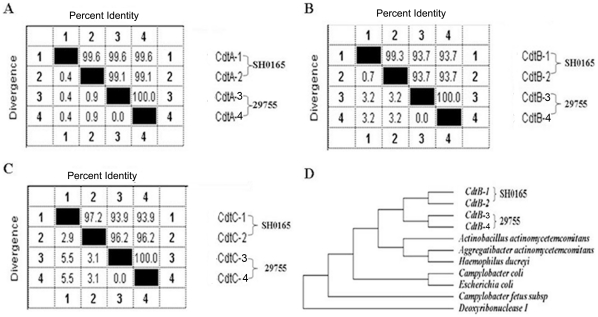
Phylogenetic analysis of two Cdts in the strain SH0165 and 29755 on the basis of the ClustalW method in Lasergene software (DNASTAR). (A), CdtA; (B), CdtB; (C), CdtC; (D), the relationship between CdtB in strain SH0165 and 29755 and that in the other bacterium species produced CdtB, on the basis of the ClustalW method.

Examination of the genome sequence of *H. parasuis* SH0165 revealed that strain SH0165 encoded two copies of Cdts (295849–297382 bp; 353207–355270 bp), which the amino acid sequences of CdtA, CdtB and CdtC showed a high (97.2%–99.6%) degree among two Cdts (Figure 1A–1C). Further analysis the sequences of Cdts in two *H. parasuis* strains, which strain SH0165 was clinically isolated in China, and strain 29755 in North American, showed that CdtB in strain 29755 only contained 63 aa, with the N-terminal 45-aa truncation and C- terminal 166-aa truncation, while CdtC contained 132 aa with the N-terminal 44-aa truncation, compared to the sequences of CdtB and CdtC in strain SH0165 (Figure S1). Phylogenetic analysis indicated that CdtB of *H. parasuis* SH0165 was clustered with strain 29755, and closed to that of *A. actinomycetemcomitans* (Figure 1D).

### Two Cdts had identical toxin activity in vitro, and CdtA and CdtB form a more active toxin than CdtB and CdtC

**Figure 2:**
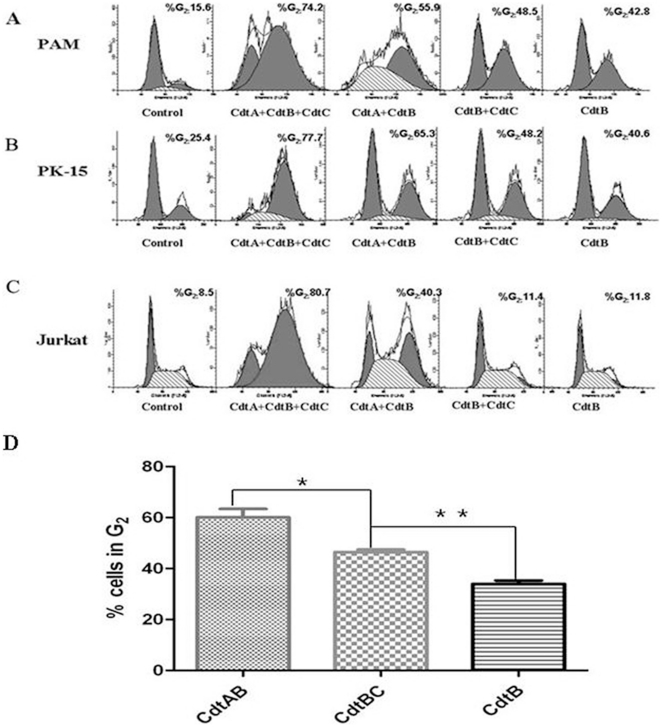
Effect of various combinations of the Cdt subunits for their ability to induce G_2_ arrest and effect of CdtA and CdtC on CdtB-induced G_2_ arrest. The cells were analyzed for cell cycle distribution by treated with Cdt proteins alone or in various combinations (as indicated) following staining with propidium iodide. (A), PAM cell; (B), PK-15 cell; (C) Jurkat cell or (D) PAM cells were exposed to 200 µl CdtB alone (50 µg/ml) or in the presence of CdtA (50 µg/ml) or CdtC (50 µg/ml). The cells were analyzed for cell cycle distribution by flow cytometry based upon propidium iodide fluorescence. The data represent the mean ± SEM of three experiments; at least 20,000 cells were analyzed per sample.

To study whether these two Cdts have toxin activity in vitro, we generated several plasmids that respectively express various *cdt* genes of two Cdts (Figure S2). These experiments showed that maximum toxin activity was observed when all three Cdt subunits were present in two Cdts (Figure 2A and 2B). The results of GST pull-down and Co-IP assays indicated that the three Cdt subunits are able to interact with one another (Figure S3). However, whether CdtA and CdtC alone or the combination with CdtA and CdtC can not induce significant cell cycle arrest (results not shown). It is noteworthy that CdtA and CdtB exhibited more toxin activity than CdtB and CdtC in *H. parasuis* (Figure 2D), which were in inconsistent with those findings in other bacterium species, which represented CdtB and CdtC exhibited more toxin activity than CdtA and CdtB.

We also tested the activity of Cdts in a broad panel of cell lines, including Jurakt, Hela, Hep-2, Vero cell lines. Among these cell lines, Jurakt cell is the most sensitive and a likely in vivo target of Cdt and further propose that Cdt represents a novel immunotoxin in other bacterium species [24]. The results indicated that these cell lines were also susceptible to the effects of Cdts, but were significantly less sensitive than PAM and PK-15 cells. It represents that the only the presence of all three Cdt peptides could induce toxin activity, except that the exposure of Jurkat cells in the presence of CdtA and CdtB also resulted in significant toxin activity, compared to in the PAM and PK-15 cells (Figure 2C).

### DNase activity associated with two Cdts

Supercoiled pET28a plasmid DNA was incubated with various concentrations of His-tagged CdtB to assess the DNase activity. Although a background level of DNase activity was observed in the control sample, significant DNase activity was observed in with CdtB incubation (Figure S4).

**Figure 3:**
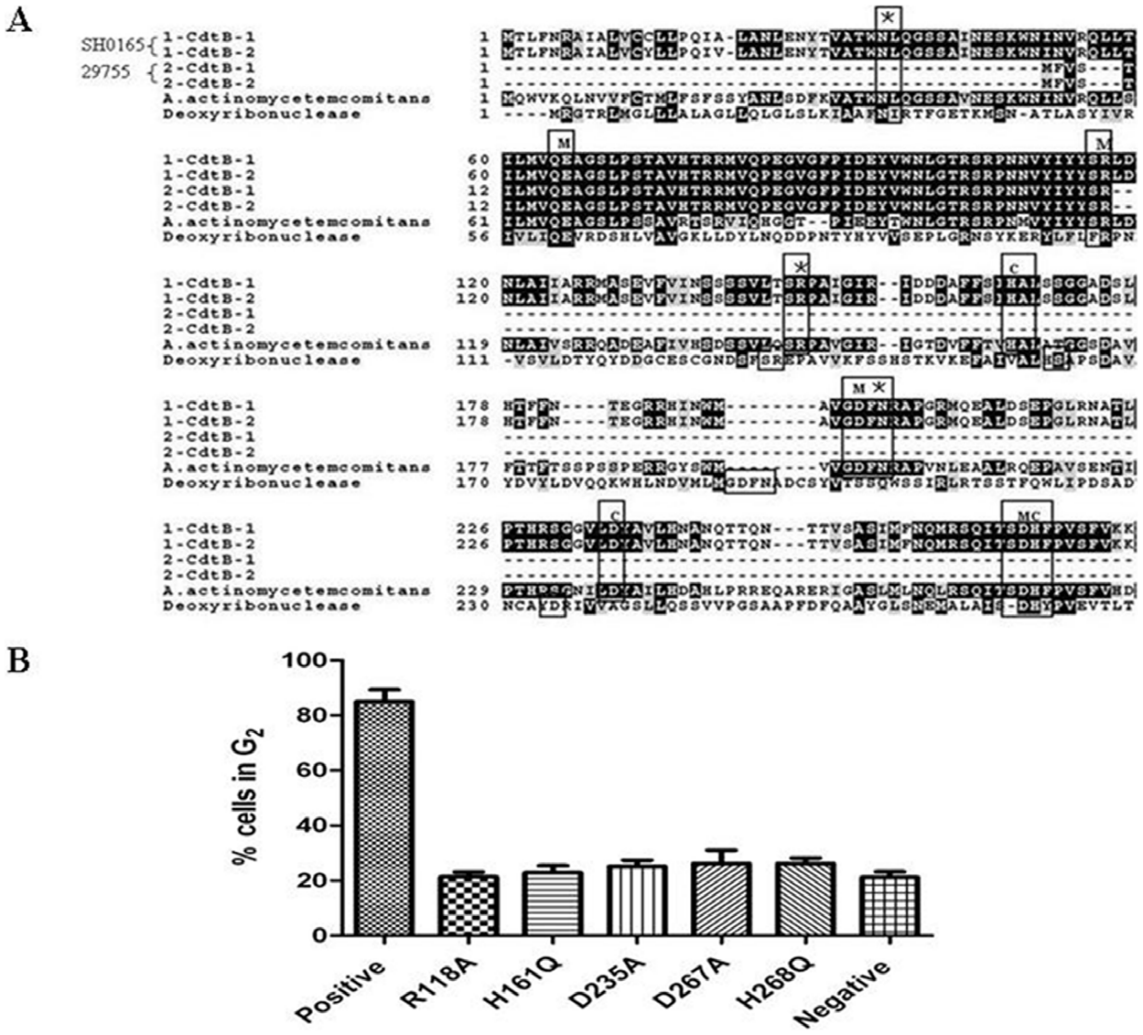
Positive-specific iterated BLAST alignment of CdtB and assessment of CdtB mutants for their ability to induce G_2_ arrest. (A), The alignment is taken directly from the final iteration. M, metal ion-binding residues; C, catalytic residues; asterisk, DNA residues. (B), PAM cells were exposed to medium alone (Negative control) or 200 µl CdtA and CdtC (50 µg/ml) in the presence of CdtB^WT^ (50 µg/ml) (Positive control) or CdtB^R118A^, CdtB^H161Q^, CdtB^D235A^, CdtB^D267A^ or CdtB^H268Q^ (50 µg/ml). Cells were analyzed for cell cycle distribution 24 h after exposure to toxin subunits using flow cytometric analysis of propidium iodide fluorescence. The numbers in each panel represent the percentages of cells in G_2_/M. Results are representative of three experiments.

Based on the pattern-specific homology that CdtB shares with mammalian type I DNase and the above observed DNase activity associated with CdtB, five CdtB mutations in amino acids corresponding to the metal ion-binding and catalytic active site of DNase I were constructed to assess the contribution to the Cdt- associated toxin activity (Figure 3A). The mutation in all five DNase-specific active site residues of CdtB combined with wild-type CdtA and CdtC failed to induce cellular distension or arrest of PAM cells (Figure 3B).

### Cdts activity was associated with the binding of Cdts to cells

**Figure 4:**
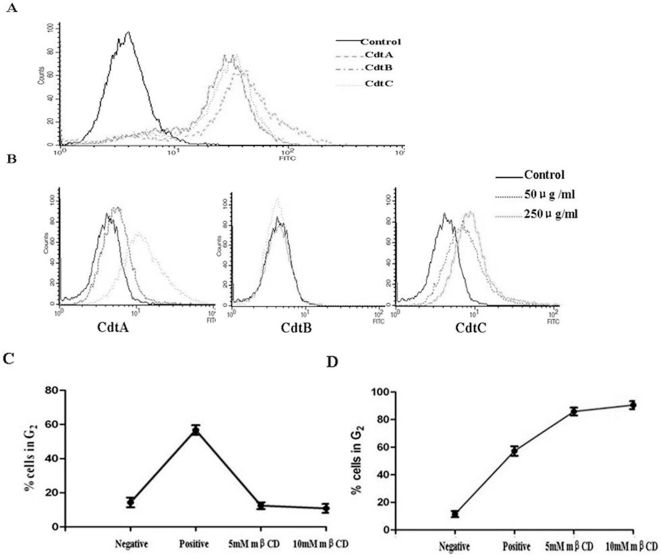
Binding of Cdt proteins to PAM and Hep-2 cells surface and effect of mβCD on Cdt-induced cell cycle arrest. (A), The PAM and Hep-2 cells were treated with the individual His-Cdt proteins for 20 min. Cells were washed three times with PBS and incubated with a murine antibody to polyhistidine for 1 h, and then after further washing the bound murine antibody was identified using a FITC-conjugated goat anti-murine F(ab′)_2_. Cells were then analysed by flow cytometry to determine the binding of the fluorophore. The cells were pre-treated with medium, 5.0 mM or 10 mM mβCD for 30 min (B) PAM cell; (C)Hep-2 cells. The cells were washed, exposed to Cdt holotoxin (50 µg/ml), incubated for 24 h, stained with propidium iodide and analysed for cell cycle distribution by flow cytometry. Numbers represent the percentage of cells in the G_2_/M phases of the cell cycle. The data represent the mean ± SEM of three experiments; 20,000 cells were analyzed for each sample.

The surface binding of the recombinant Cdt proteins to various cells was determined using flow cytometry. The results showed that all the three Cdt subunits were able to bind cell membrane in PAM cell, and the ability of surface binding was: CdtA>CdtC>CdtB (Figure 4A). However, this study failed to demonstrate any binding of CdtB in other tested cell lines, for example Hep-2 cells even when added to cells at concentrations as high as 250 µg/ml. In contract, the binding of both CdtA and CdtC was dose dependent (Figure 4B). The surface binding ability of CdtA was slightly stronger than CdtC at concentrations as high as 250 µg/ml.

To further test whether Cdt intoxication were dependent on the presence of cholesterol, the cells were exposed to 5 mM and 10 mM mβCD for 30 min, before the intoxication was measured. Fifty-two percent of PAM cells exposed to Cdt in the absence of mβCD were arrested in the G_2_ phage, while a dose-dependent reduction of G_2_ population from 9.8% to 6.4% was observed upon intoxication of 5 mM and 10 mM mβCD-treated cells, respectively (Figure 4C). Interestingly, in contrast to PAM cells, the percentage of G_2_ population was increased upon intoxication of 5 mM and 10 mM mβCD-treated Hep-2/Vero cells respectively, compared to the cells exposed to Cdt in the absece of mβCD (Figure 4D). It is noteworthy that this is the first report that cells treated with mβCD could enhance the toxin activity rather than reduce the toxin activity. Parallel experiments verified that mβCD did not adversely affect the tested cells viability as determined by propidium iodide exclusion.

### The CdtC subunit contains an atypical CRAC region

**Figure 5:**
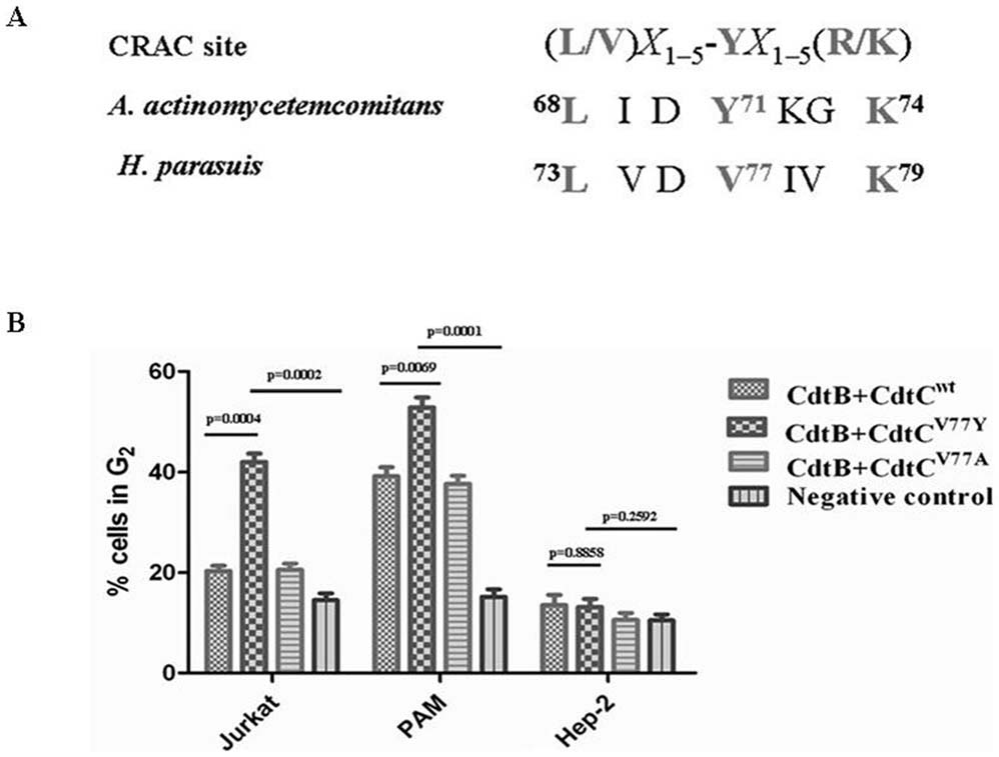
Structural alignment and assessment of CRAC site mutants in CdtC subunits for their ability to induce G_2_ arrest. (A), structural alignment of CRAC site in *H. parasuis*, CdtC in *A. actinomycetemcomitans* and the classical CRAC sites. (B), assessment of CRAC site mutants for their ability to induce G_2_ arrest. The cells were incubated with medium alone, 50 µg/ml CdtABC^WT^, 50 µg/ml CdtABC^V77Y^ or 50 µg/ml CdtABC^V77A^ for 24 h, stained with propidium iodide, and analyzed for cell cycle distribution by flow cytometry as described above.

Collectively, our results strongly support the notion that Cdt holotoxin interaction with membranes and is dependent upon cholesterol. Motif analysis of CdtC subunit identified an atypical CRAC region, ^73^LVDV^77^IVK^79^, with 77 site amino acid (V) instead of 71 site amino acid (Y) of CdtC of *A. actinomycetemcomitans* (Figure 5A). To determine whether this point mutation of CRAC site was affected the toxin activity of Cdt, two single-point mutant, CdtC^V77Y^ and CdtC^V77A^ were generated and assessed for its effect to induce G_2_ arrest in various cell lines. Twenty-four hours after exposure to CdtB and CdtC^V77Y^, an increase in the percentage of G_2_ cells (42.1%) was observed in Jurkat cells; compared to treated with CdtB and CdtC^wt^ (20.3%) or CdtB and CdtC^V77A^ (20.6%). Although CdtB and CdtC^wt^ could induce cell arrest in PAM cells (39.2%), CdtB and CdtC^V77Y^ significantly enhanced the cell toxicity (52.9%) compared to CdtB and CdtC^wt^. However, the ability to induce G_2_ arrest of these CRAC mutants was not obvious change in the Hep-2 cells (Figure 5B).

### Cdt is a conservative putative virulence factor for *H. parasuis*

**Figure 6:**
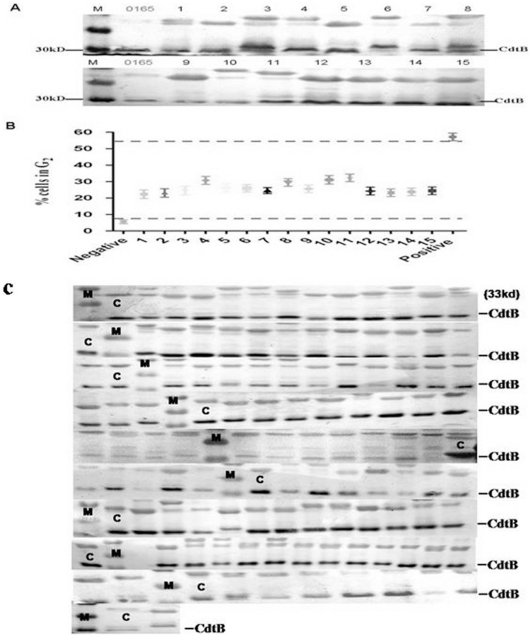
Detection of the expression and Cdt activity of CdtB from 15 *H. parasuis* reference strains and 109 *H. parasuis* clinical isolates. (A), Whole cell proteins of 15 *H. parasuis* reference strains were applied to western bolt and detected by anti-CdtB specific antibody.1–15 represents 15 *H. parasuis* reference strains, 0165 strain was used as positive control. (B), Cells were incubated with medium alone (negative control), 50 µg/ml CdtABC^WT^ or 200 µg/ml whole cell proteins of 15 *H. parasuis* reference strains for 24 h, stained with propidium iodide, and analyzed for cell cycle distribution by flow cytometry as described above. (C), the whole cell proteins of 109 clinical isolates were applied to western bolt and detected by anti-CdtB specific antibody. M, marker; C, 0165 strain was used as positive control.

Eventually, we also investigated the expression of CdtB in 15 *H. parasuis* reference serotypes. The results showed all 15 *H. parasuis* reference strains expressed the CdtB protein (Figure 6A) and demonstrated Cdt activity (Figure 6B). Additionally, a series of 109 clinical isolates were test for the expression of the CdtB protein by western blot, as well as for production of a Cdt cytopathic effect. All the tested strains expressed the CdtB protein (Figure 6C), also produced a CDT cytophic effect and cell-cycle arrest (results not shown).

## Discussion

The co-evolution of bacterial pathogens and their hosts has contributed to the development of very complex and sophisticated functional pathogen-host interactions. Thus, well-adapted pathogens have evolved a variety of strategies to manipulate host cell functions precisely. For example, a group of unrelated Gram-negative pathogenic bacteria have evolved a toxin, known as cytolethal distending toxin that has the ability to control cell cycle progression in eukaryotic cells [27]. However, the molecular and cellular mechanism of Cdt host interactions in *H. parasuis* have never been identified.

In this study, we identified that *H. parasuis* SH0165 encodes two copies of Cdts, and show as the uniform function in vitro. Compared to the other bacterium species only produced one Cdts, the co-evolution of *H. parasuis* and their hosts has produced more than one Cdts to better manipulate host cell functions, indicating that the virulence may became stronger in the process of the evolution of *H. parasuis*. On the other hand, the sequences of CdtB and CdtC showed the obvious inconsistency between strain SH0165 and 29755, although both these two *H. parasuis* strains are serotype 5. These results indicated that a considerable genetic heterogeneity of Cdts were present within the same serovars.

Those bacterial species that exhibit Cdt activity have proved to be recalcitrant to the isolation and purification of the individual Cdt proteins. One of the major difficulties in Cdt protein purification from the native bacterial species is a consequence of the synthesis of very small quantities of highly active gene products. To overcome these obstacles, studies of the composition and organization of the CDT holotoxin have been performed using recombinant proteins isolated from clones that contain single cdt genes. All known Cdt operons contain three genes, *cdtA*, *cdtB*, and *cdtC*, encoding proteins with similar molecular masses (20–35 kDa). However, there are conflicting reports regarding whether a single gene of multiple *cdt* genes encode the holotoxin responsible for the induction of cell cycle arrest in target cell. Jorge E. Gala'n [28] showed *S. typhi cdtB* encodes a functional protein and that, despite the absence of CdtA and CdtC, this bacterium produces a *cdtB*-dependent Cdt activity that requires bacterial internalization into host cells. In contrast, Stevens et al. [29] reported that *cdtC* encodes the structural toxin of *H. ducreyi*. Our results demonstrated that CdtB alone was sufficient to induce PAM/PK cells to undergo G_2_ arrest, and maximum toxin activity was dependent upon the availability of all three *cdt* genes, which are consistent with observations on the Cdt toxins of other bacterium species. It is noteworthy that CdtA and CdtB exhibited more toxin activity than CdtB and CdtC in *H. parasuis*, suggesting that CdtA and CdtB form a more active toxin than CdtB and CdtC. These results were in inconsistent with that findings in *A. actinomycetemcomitans*, which represented CdtB and CdtC exhibited twice the activity of CdtA and CdtB [30]. Using the position-specific iterated BLAST program, we identified a distant, but significant, relationship between CdtB of *H. parasuis* and DNase I homologues of mammalian origin. The results provided strong evidence that the DNase sequence homology observed in CdtB is functionally conserved, and the DNase activity associated with CdtB is required for the Cdt-mediated cell cycle arrest [31].

Although the biological activity of Cdts is due to the DNase activity of CdtB, the presence of a nuclear localization signal in CdtB of *A. actinomycetemcomitans* also supports the hypothesis that DNA is the molecular target [32]. However this could not rule out the possibility that CdtB activity may have an effect on other signaling pathways as well. Cdt has been recently reported to enter cells via clathrin-coated pits, which is consistent with a receptor-mediated process [22]. If CdtB is the active moiety responsible for cell cycle arrest, it must enter the cell in order to damage the DNA. Our results showed that all three Cdt subunit could able to bind to PAM cells, while only CdtA and CdtC, not CdtB could bind to Hep-2 cells, which is the classic mode of cell entry of many bacterial A/B toxins, which the B domain binds to the cell surface receptor allowing the uptake of the toxin A domain [33]. From the surface binding results of different cell lines, there seems to have a balance relationship between whether CdtB can bind to the cells and induce cell toxicity. However, CdtB alone was sufficient to induce cell arrest in PAM cells, indicating that CdtB alone are able to enter into cells without CdtA and CdtC by other modulation of signaling pathways.

The binding of Cdt to the plasma membrane of target cells has recently been well documented by Lee et. al [34]. An increasing number of bacterial toxin, such as cholera toxin, *Aeromonas hydrophila* aerolysin, *Clostridium* perfringenslota-toxin and *Helicobacter pylori* VacA, have been shown to interact with microdomains in the plasma membrane, known as lipid rafts [35]–[37]. These domains are enriched in cholesterol, sphingolipids and glycosylphosphatidylinositol (GPI)-anchored proteins, and their integrity can be disrupted by drugs which can extract cholesterol from the plasma membrane, such as mβCD. In line with these observations, our results showed the treatment of PAM and PK-15 cells with mβCD abolished cellular intoxication, indicating that cholesterol depletion protected cells from the ability of the Cdt holotoxin to induce G_2_ arrest. It is surprising that Hep-2 and Vero cells, treated with mβCD did not abolished cellular intoxication, but enhanced the cellular intoxication, indicating that the cellular intoxication might be mediated by another action of binding of Cdt to the plasma membrane in these cell lines.

Our previous studies clearly indicated that the Cdt holotoxin interacts with the PAM cell surface and specifically associated with cholesterol. Several proteins have been shown to bind to cholesterol; including the benzodiazepine receptor, the human immunodeficiency virus transmembrane protein gp41, and the caveolin [24]. Each of these cholesterol-binding proteins contains the cholesterol recognition amino acid consensus sequence (CRAC), L/V)X_1–5_-YX_1–5_(R/K) in which X_1–5_ represents between one and five residues of any amino acid. Based upon the results from our experiments along with the effects of cholesterol depletion on Cdt toxicity, we identified an atypical CARC within CdtC subunit, ^73^LVDV^77^IVK^79^, which the 77 site residue (V) instead of the tyrosine residue (Y) in the classical CRAC site. Our results showed CdtB and CdtC^V71Y^ could significantly enhance cell toxicity in in Jurkat and PAM cells, compared with CdtB and CdtC^wt^. This may be one of the reasons that CdtB and CdtC^wt^ could not induce cell arrest in Jurkat cells, and CdtA and CdtB form a more active toxin than CdtB and CdtC. It should be noted that the tyrosine residue (Y) in this position has been shown by mutational analysis to be critical for cholesterol binding in other proteins [24]. These observations indicated that the mutation of the tyrosine residue within this motif may result in a loss of cholesterol binding, thereby decrease cell toxicity, which suggests that the evolution of Cdt in *H. parasuis* was toward the direction of weakened virulence. However, further studies are necessary to unravel the functions of this complex evolution relationship in the physiology and pathogenicity of *H. parasuis*.

Rapidly growing cells, such as epithelial cells, monocytes, and lymphocytes, cannot proliferate when treated with Cdt [38], leading to the theory that Cdt damages epithelial cells in the mucosa and also may modulate the immune system by preventing proliferation of macrophages and lymphocytes. *C. jejuni* Cdt has been shown to mediate the release of interleukin- 8 from INT407 cells [39], suggesting that induction of proinflammatory cytokines may be elicited by the toxin. Our results showed all the 15 *H. parasuis* reference strains and 109 clinical isolates expressed the CdtB protein and demonstrated Cdt activity, indicating that Cdt is conservative virulence factor for *H. parasuis*. However, there are no genetic operating systems to study *H. parasuis* infection in vivo, nor are there any in vivo models for discerning the role that Cdt plays in disease. With the development of genetic models, the role that Cdt plays in the pathogenesis of *H. parasuis* may be determined.

In conclusion, our results contribute to a better understanding of the Cdt mode of action and highlight some unique aspects of the biology of this bacterial toxin family in *H. parasuis*. For example, *H. parasuis* encodes two copies of cytolethal distending toxins (Cdts), which these two Cdts showed the uniform toxin activity in vitro. We demonstrate that three Cdt peptides can form an active tripartite holotoxin that exhibits maximum cellular toxicity, and CdtA and CdtB form a more active toxin than CdtB and CdtC. Moreover, the cellular toxicity is associated with the binding of Cdt subunits to cells. Further analysis indicates that CdtC subunit contains an atypical cholesterol recognition/interaction amino acid consensus (CRAC) region. The mutation of CRAC site resulted in decreased cell toxicity. Finally, western blot analysis show all the 15 *H. parasuis* reference strains and 109 clinical isolates expressed CdtB subunit and demonstrated Cdt activity, indicating that Cdt is a conservative putative virulence factor for *H. parasuis*.

## Supporting Information

Figure S1 **Sequence analysis of two Cdts (CdtA, CdtB and CdtC) in the strain SH0165 and 29755.**
(TIF)

Figure S2 **SDS–PAGE and Western blot analysis of purified recombinant His6-tagged Cdt proteins of two Cdts.** 6 µg of each protein sample was applied to the gel and stained with CBB (A). The blot was probed with His-Tag monoclonal antibody at a 1∶3000 dilution and horseradish peroxidase-conjugated anti-mouse IgG diluted 1∶3000. Immunopositive bands were detected bychemiluminescence (B).
(TIF)

Figure S3 **GST pulldown and Co-immunoprecipitation assays for Cdt protein interactions.** (A), GST pulldown assays for Cdt protein interactions. His-tagged CdtA, CdtB, or CdtC proteins were probed with purified GST-CdtB, or GST (as a negative control). Cdt proteins bound to the glutathione-agarose beads in the samples were detected by Western blotting with a monoclonal antibody directed to the His epitope tag. (B), co-immunoprecipitation assays for Cdt protein interactions. His -tagged Cdt proteins in combination with GST-Cdt proteins (as indicated at the top of each panel) were subjected to co-immunoprecipitation assays with rabbit polyclonal antibodies specific to different Cdt proteins (as indicated below each panel). His-tagged Cdt proteins bound to the protein A-Sepharose beads in the samples were detected by Western blotting with a monoclonal antibody directed to the His epitope tag.
(TIF)

Figure S4 **DNase activity of recombinant His-tagged CdtB.** Purified His-tagged CdtB (0–8 µg) was incubated for 1 h at 37°C with supercoiled plasmid DNA. The contents of the reaction tubes were applied to a 1% agarose gel. The gel was stained with ethidium bromide. S, supercoiled form of the plasmid DNA; R, relaxed form of the plasmid DNA.
(TIF)

Table S1 ***H. parasuis*** **two Cdts plasmid constructs.**
(DOC)

Table S2 ***H. parasuis*** **CdtB and CdtC mutant constructs.**
(DOC)
