# Supplementary figures and images for: Haemophilus parasuis Encodes Two Functional Cytolethal Distending Toxins: CdtC Contains an Atypical Cholesterol Recognition/Interaction Region
Source: PLoS One. 2012 Mar 7;7(3):e32580. doi: 10.1371/journal.pone.0032580 (PMC3296717; doi:10.1371/journal.pone.0032580)

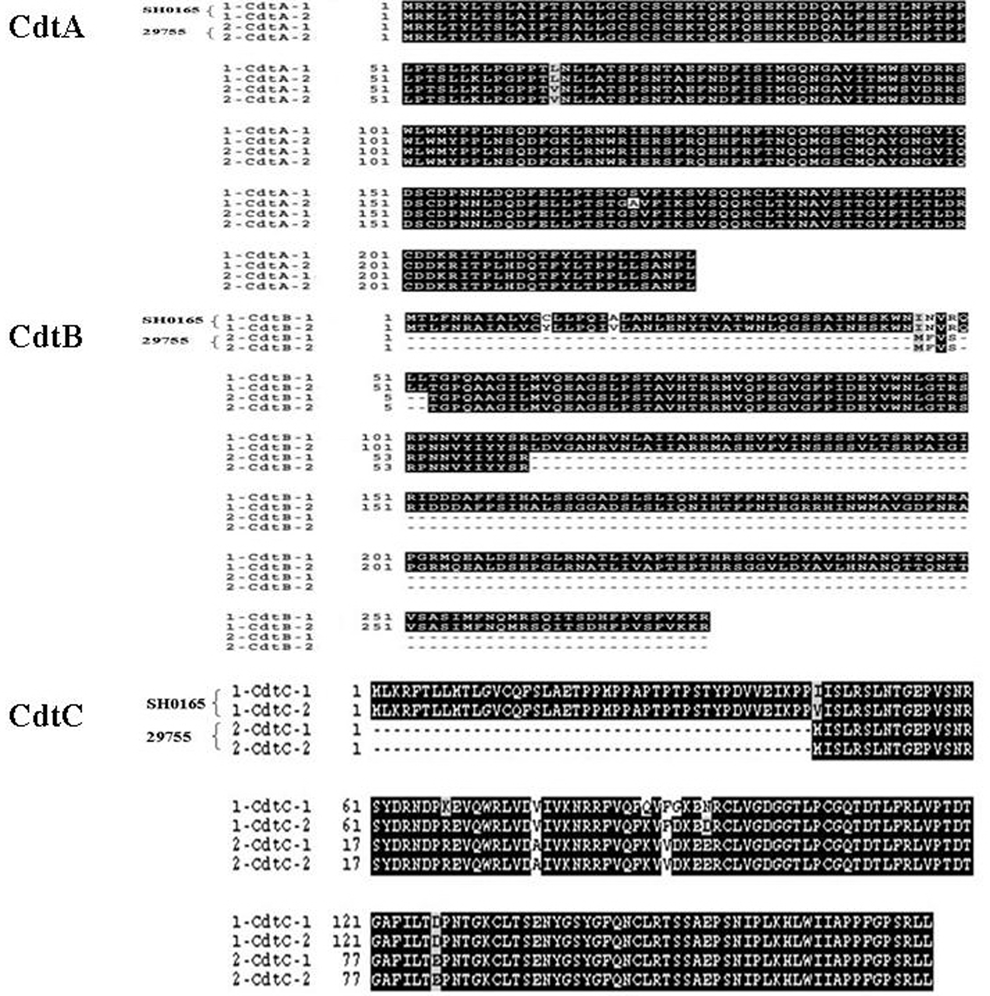

Supplement: Figure S1 — Sequence analysis of two Cdts (CdtA, CdtB and CdtC) in the strain SH0165 and 29755. (TIF) [file pone.0032580.s001.tif]

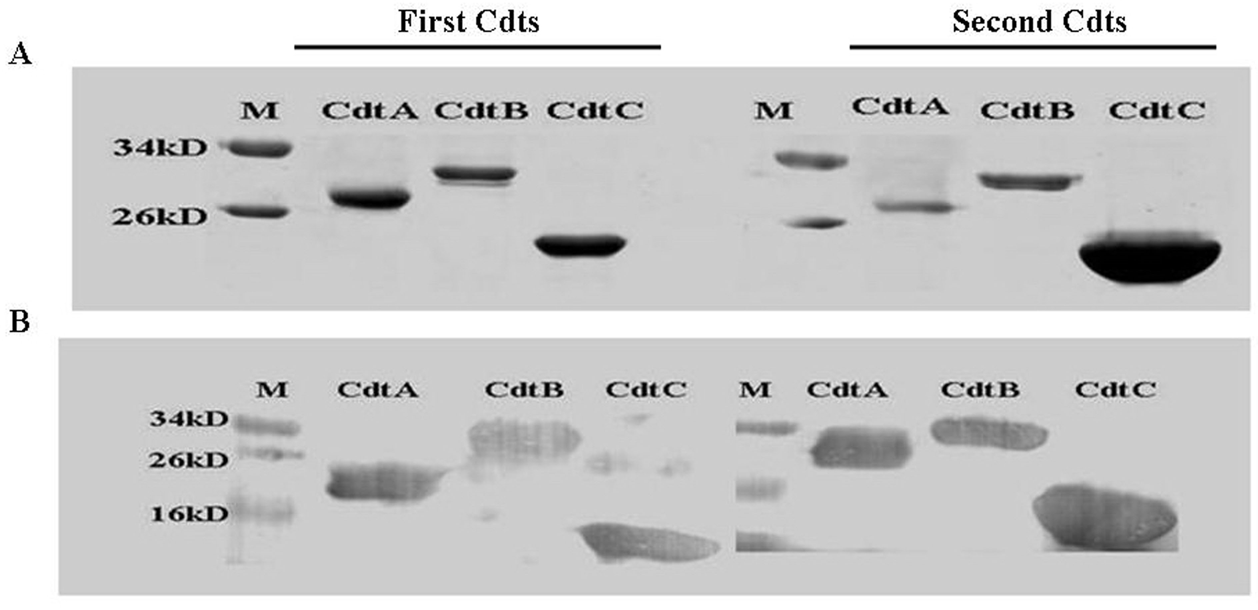

Supplement: Figure S2 — SDS–PAGE and Western blot analysis of purified recombinant His6-tagged Cdt proteins of two Cdts. 6 µg of each protein sample was applied to the gel and stained with CBB (A). The blot was probed with His-Tag monoclonal antibody at a 1∶3000 dilution and horseradish peroxidase-conjugated anti-mouse IgG diluted 1∶3000. Immunopositive bands were detected bychemiluminescence (B). (TIF) [file pone.0032580.s002.tif]

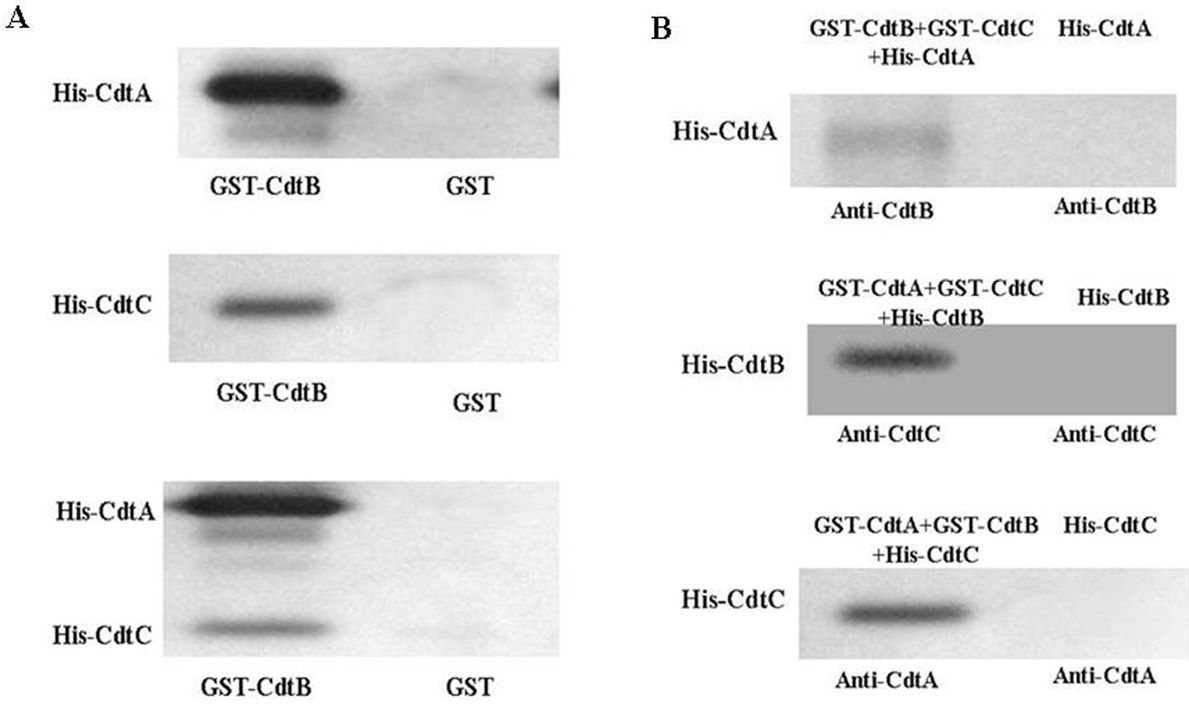

Supplement: Figure S3 — GST pulldown and Co-immunoprecipitation assays for Cdt protein interactions. (A), GST pulldown assays for Cdt protein interactions. His-tagged CdtA, CdtB, or CdtC proteins were probed with purified GST-CdtB, or GST (as a negative control). Cdt proteins bound to the glutathione-agarose beads in the samples were detected by Western blotting with a monoclonal antibody directed to the His epitope tag. (B), co-immunoprecipitation assays for Cdt protein interactions. His -tagged Cdt proteins in combination with GST-Cdt proteins (as indicated at the top of each panel) were subjected to co-immunoprecipitation assays with rabbit polyclonal antibodies specific to different Cdt proteins (as indicated below each panel). His-tagged Cdt proteins bound to the protein A-Sepharose beads in the samples were detected by Western blotting with a monoclonal antibody directed to the His epitope tag. (TIF) [file pone.0032580.s003.tif]

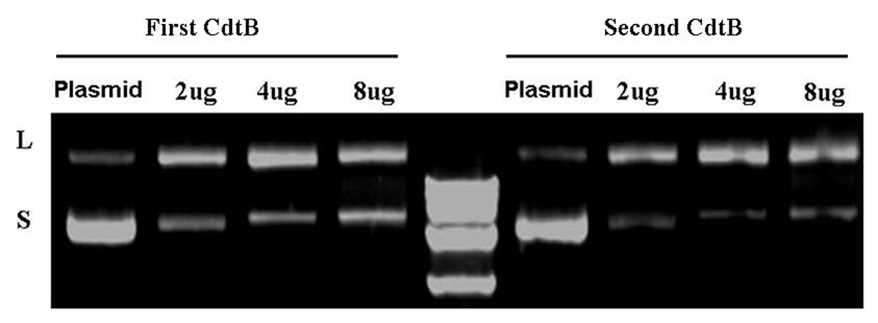

Supplement: Figure S4 — DNase activity of recombinant His-tagged CdtB. Purified His-tagged CdtB (0–8 µg) was incubated for 1 h at 37°C with supercoiled plasmid DNA. The contents of the reaction tubes were applied to a 1% agarose gel. The gel was stained with ethidium bromide. S, supercoiled form of the plasmid DNA; R, relaxed form of the plasmid DNA. (TIF) [file pone.0032580.s004.tif]
