# Supplementary material for: Haemophilus parasuis Encodes Two Functional Cytolethal Distending Toxins: CdtC Contains an Atypical Cholesterol Recognition/Interaction Region
Source: PLoS One. 2012 Mar 7;7(3):e32580. doi: 10.1371/journal.pone.0032580 (PMC3296717; doi:10.1371/journal.pone.0032580)
Supplement: Table S2 — H. parasuis CdtB and CdtC mutant constructs. (DOC) [file pone.0032580.s006.doc]

**Table 2.  *H. parasuis* CdtB and CdtC mutant constructs**

| Plasmid | Primer | Sequence |
| --- | --- | --- |
| pET28aCdtB^R118A^ | P11  P12 | Gggggcaaatgcagttaatcttg  tgcatttgcccccacatcaaga |
| pET28aCdtB^H161Q^ | P13  P14 | ctttagtattcaggctctttcatctggagg  ctgaatactaaagaaagcatcgtcatcaa |
| pET28aCdtB^D235A^ | P15  P16 | tggagtattggcatatgcagtgttac  tgccaatactccaccagaacgatg |
| pET28aCdtB^D267A^ | P17  P18 | gattacctccgcacatttcccagtc  tgcggaggtaatctgcgatctc |
| pET28aCdtB^H268Q^ | P19  P20 | tacctccgatcagttcccagtcagcttt  ctgatcggaggtaatctgcgatctc |
| pET28aCdtC^V77Y^ | P21  P22 | acttgttgattatattgttaaaaatcgtc  acgatcaacaagtcgccactgc |
| pET28aCdtC^V77A^ | P23  P24 | Acttgttgatgcaattgttaaaaatcgtc  catcaacaagtcgccactgcac |
